# Prenatal multiple micronutrient supplementation is associated with improved maternal gestational weight gain: A prospective longitudinal study in Parepare, Indonesia

**DOI:** 10.1002/ijgo.70389

**Published:** 2025-07-19

**Authors:** Sabaria Manti Battung, Eline M. van der Beek, Henk Groen

**Affiliations:** ^1^ Department of Pediatrics University Medical Center Groningen, University of Groningen Groningen The Netherlands; ^2^ Department of Nutrition, Faculty of Public Health Hasanuddin University Makassar Indonesia; ^3^ Nestlé Institute of Health Sciences Nestlé Research, Societé du Produits Nestlé Lausanne Switzerland; ^4^ Department of Epidemiology, University Medical Center Groningen University of Groningen Groningen The Netherlands

**Keywords:** body mass index, gestational weight gain, Indonesia, multiple micronutrient supplementation, pregnant women

## Abstract

**Objective:**

The aim of the present study was to assess the association between prenatal multiple micronutrient supplementation (MMS) intake levels and gestational weight gain (GWG) recommendations in pregnant women in Indonesia.

**Methods:**

We performed a population‐based prospective cohort data collection during the rollout of MMS supplementation in Parepare, Indonesia. A total of 1216 pregnant women were enrolled and followed up until after delivery. Data on pre‐pregnancy weight, height, and maternal parity were collected, as well as the starting trimester of MMS consumption and total number of tablets consumed. Weight measurements were performed at enrollment and in the late third trimester. The threshold applied for MMS adherence was ≥90 tablets. The Institute of Medicine 2009 guideline was used to determine GWG adequacy. Associations between total MMS consumption and GWG were assessed by binary logistic regression.

**Results:**

Women with underweight, normal weight and overweight/obesity accounted for 11.4%, 58.1%, and 30.5% of the cohort, respectively; overall, 63.2% had inadequate, 26.3% had adequate, and 10.5% had excessive GWG; 53.9% consumed ≥90 MMS tablets. Crude analysis showed that women who consumed ≥90 MMS tablets had higher odds of achieving adequate GWG (OR = 1.34, 95% CI: 1.05–1.69). Several potential confounders were examined but did not materially affect the association.

**Conclusion:**

Our findings indicate that many women in Indonesia do not achieve adequate GWG. Ensuring the recommended MMS dose during pregnancy may improve nutrition status and increase the likelihood of adequate GWG. Thus, advice on the provision of MMS consumption and appropriate GWG should be part of antenatal care services for all pregnant women.

## 1 INTRODUCTION

The double burden of malnutrition remains a significant health problem in low and middle‐income countries (LMICs).^1^ While the prevalence of underweight in women aged 20–49 years has decreased from 11.6% in 2000 to 9.7% in 2016, the prevalence of overweight and obesity has shown an increase, rising from 31.7% to 39.2%.^2^ Both underweight and overweight/obesity can lead to micronutrient deficiencies, either due to the inadequate intake of nutrient‐rich food or by consuming energy‐dense but nutrient‐poor foods.^3^ Globally, a considerable percentage (69%) of women of reproductive age suffer from one or more micronutrient deficiencies (iron, zinc and folate) of which about 57% live in Southeast Asia and the Pacific region.^4^ Iron‐deficiency anemia is the most common micronutrient deficiency, affecting around 37% of pregnant women.^5^

Good nutritional status prior to pregnancy has been shown to contribute to adequate gestational weight gain.^6^ Given the increased micronutrient needs during pregnancy driven by the changes in maternal physiology and the needs related to placenta and fetal development, suboptimal nutrient status may also increase the risk of micronutrient deficiencies during pregnancy.^7^ Pre‐pregnancy body mass index showed a strong correlation with gestational weight gain that fell outside the recommended weight gain.^8^ Failure to meet the recommended guidelines for gestational weight gain (GWG), either by gaining less or more weight, increases the risk of adverse maternal and pregnancy outcomes.^9^ Inadequate GWG has been reported to be quite prevalent among Asian populations.^10^, ^11^, ^12^ A systematic review and meta‐analysis that included data across continents and ethnicities revealed that 31% of Asian women had GWG below guidelines.^13^ Similarly, a meta‐analysis of data from diverse international cohorts indicated that nearly 23% of pregnant women had GWG below guidelines. In the same analysis, 47% had weight gain above the guidelines.^9^

The WHO has recommended worldwide supplementation of specific micronutrients to improve nutrient status and reduce adverse pregnancy outcomes for both mother and child, helping to achieve the SDG 2 and 3 goals.^14^ Provision of prenatal multiple micronutrient supplementation (UNIMMAP‐MMS), consisting of 15 vitamins and minerals, has consistently shown more benefits than iron folic acid supplementation in reducing the risk of preterm birth, small for gestational age and low birth weight.^15^ These studies predominantly focused on comparing the effect of the different supplements on birth outcomes through randomized controlled trials.

There are to date, limited studies reporting the effect of multiple micronutrient supplementation on gestational weight gain. A recent meta‐analysis using data from several previous randomized controlled trials (RCT's) in LMIC's countries found that provision of multiple micronutrient supplementation (MMS) during pregnancy led to higher gestational weight gain compared to iron and folic acid supplementation.^16^ These clinical studies achieved a relatively high intake of MMS (compliance >80%). However, there are limited studies investigating the association between MMS consumption and GWG in a community‐based setting. Furthermore, data on GWG in Indonesian populations is scarce. The present study aimed to assess the association between MMS and weight gain adequacy among Indonesian pregnant women in standard antenatal care setting.

## 2 MATERIALS AND METHODS

### 2.1 Study design and participants

This observational longitudinal cohort study was set up in Parepare, South Sulawesi province, Indonesia.^17^ Parepare is a small town with a population density of 1.467 people/km^2^ and consisting of eight community health center facilities (Puskesmas). Puskesmas provides primary healthcare, referral services, and community support. It is staffed with practitioners, dentists, nurses, midwives, pharmacists, nutritionists, and analysts. Midwives provide antenatal care services and record pregnancy information in the Maternal and Child Health Book (MCHB). The MMS program has been rolled out since January 2021 to replace the standard care IFA supplementation and was integrated into the ANC program at community health centers.

The start of our data collection coincided with the start of the implementation of MMS supplementation across the district. Hence, selection of participants was done in two ways: (1) pregnant women who were provided and willing to consume MMS were recruited by asking them directly about their willingness to participate in the data collection during the first ANC visit, and (2) those who had started MMS before inclusion as part of standard antenatal care were identified by checking their records of MMS consumption in the MCHB and validation against the remaining tablets in the bottle. All participants were followed up from enrolment until delivery. The study information was provided, and written informed consent was obtained from each participant.

The sample size for the cohort was based on an expected difference in MMS consumption between low birthweight and normal weight infants, which was the primary outcome.^17^ The study assumed a 5% prevalence of low birth weight and an effect size of 0.4–0.5 for MMS consumption. To achieve an 80% power to detect the effect size with a two‐sided alpha level of 5%, indicating reduced MMS exposure in low birth weight infants, a minimum of 1260 mother‐infant pairs was set, estimating inclusion of 60 low birth weight and 1200 normal weight infants. Overall, 1216 pregnant women were enrolled in the study and followed until after delivery.

### 2.2 Data collection

Data were collected by two trained midwives who were assigned to each community healthcare facility. Data on maternal characteristics, pre‐pregnancy weight, height, and maternal parity were collected, as well as a starting trimester of MMS consumption and total number of tablets consumed. These data were obtained through survey administered questionnaire, and entered into an electronic format, with a secure database constructed using REDCap (Research Electronic Data Capture, https://www.project‐redcap.org). Data on pre‐pregnancy weight was self‐reported. Height was measured during the initial prenatal ANC visit. Gestational age was determined based on the last menstrual period (LMP) reported.

The MMS tablets were distributed during regular ANC visits. Pregnant women were provided with 30 tablets in a bottle at each visit and instructed to take one per day. They were also asked to return the bottle for refilling with another 30 tablets at the subsequent visits. The total number of MMS consumed was calculated based on the missing tablets from the bottle. Midwives then verified the number of tablets consumed at each subsequent visit. The number of MMS consumed was categorized as <90 tablets and ≥90 tablets. This cutoff was based on the IFA policy in Indonesia.^18^

### 2.3 Standard measurement

Seca 877 scale was used to measure the weight and wall‐mounted measuring tape microtoise as 200 cm to measure the height of women. They were measured in light clothes and without shoes on.

Body mass index (BMI, calculated as weight in kilograms divided by the square of height in meters) was calculated and classified women as underweight (BMI <18.5), normal weight (18.5–24.9), overweight (25.0–29.9) and obesity (≥30) based on the WHO criteria. Total GWG during pregnancy was calculated by subtracting pre‐pregnancy weight from the final weight measurement in the third trimester (30–41 weeks). This total GWG was then compared with IOM recommended weight increase to determine GWG adequacy. The IOM classifies recommended GWG based on pre‐pregnancy BMI as follows: underweight 12.5–18 kg, normal weight 11.5–16.0 kg, overweight 7–11.5 kg, and obese 5–9 kg.^6^

### 2.4 Statistical analysis

Data were analyzed using SPPS Statistics for windows version 28.0. Characteristics of participants were summarized using proportions. We analyzed the GWG adequacy based on three categories, which were inadequate, adequate, and excessive. This was then simplified into two categories, by combining the adequate and excessive categories, to allow analysis by logistic regression and ease interpretation. Univariable and multivariable logistic regression were conducted to identify the association between the number of MMS consumed and GWG adequacy, reporting the odds ratio (OR) and 95% confidence intervals (CI). Statistical significance was determined using *P* values less than 0.05 as the threshold. Cubic spline regression was performed using R‐project package R‐3.3.3 for Windows to explore possible non‐linearity of the relationship between the total number of MMS consumed and the probability of adequate GWG.

### 2.5 Ethics approval

Ethical approval was obtained from the University of Hasanuddin's Ethical Committee (7267/UN4.14.1/TP.01.02/2021) before the start of inclusion.

## 3 RESULTS

**FIGURE 1:**
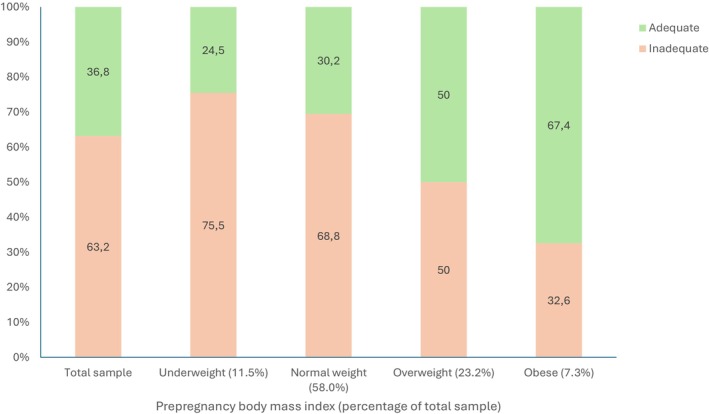
Prevalence of gestational weight gain adequacy by pre‐pregnancy body mass index (BMI, calculated as weight in kilograms divided by the square of height in meters).

A total of 1216 pregnant women were included in this analysis. Most participants (63.2%) had inadequate GWG, 26.3% had adequate GWG and 10.5% had excessive GWG according to the IOM classification. The proportion of GWG adequacy varied based on pre‐pregnancy BMI status (Figure 1). Yet, women who started pregnancy underweight had the highest percentage of inadequate GWG (75.5%), and obese women showed the lowest percentage (32.6%).

Characteristics of participants are shown in Table 1. Most women were typically aged ≥30 (36.6%), started taking MMS in the first trimester (53.9%), had no anemia (92.0%), and had ≥5 dietary diversity scores (83.2%). Significant differences between women with inadequate and adequate weight gain were seen in education, occupation, pre‐pregnancy BMI, gravidity, maternal height, and smoking exposure (*P* < 0.05).

**TABLE 1:** Maternal characteristics according to gestational weight gain adequacy.

Variables	*N* (%)	GWG adequacy		*P* value^a^
		Inadequate *n* (%)	Adequate *n* (%)	
Total	1216	768 (63.2)	448 (36.8)	
Age category (years)				
<20	72 (5.9)	47 (6.1)	25 (5.6)	0.22
20 to <25	280 (23.0)	175 (22.6)	105 (23.4)	
25 to <30	420 (34.5)	251 (32.7)	169 (37.7)	
≥30	444 (36.6)	295 (38.6)	149 (33.3)	
Education				
Junior high school	136 (11.2)	91 (11.8)	45 (10.0)	**0.03**
Senior high school	222 (18.3)	154 (20.1)	68 (15.2)	
Diploma	494 (40.6)	312 (40.6)	182 (40.6)	
University	364 (29.9)	211 (27.5)	153 (34.2)	
Occupation				
Not working	973 (80)	628 (81.8)	345 (77.0)	**0.04**
Working	243 (20.0)	140 (18.2)	103 (23.0)	
Pre‐pregnancy BMI				
Underweight (<18.5)	139 (11.4)	105 (13.7)	34 (7.6)	**<0.001**.
Normal (18.5 to <25)	706 (58.1)	493 (64.2)	213 (47.5)	
Overweight (25 to <30)	282 (23.2)	141 (18.4)	141 (31.5)	
Obesity (>30)	89 (7.3)	29 (3.8)	60 (13.4)	
Time of MMS introduction				
First trimester	655 (53.9)	413 (53.8)	242 (54.0)	0.70
Second trimester	501 (41.2)	320 (41.7)	181 (40.4)	
Third trimester	60 (4.9)	35 (4.6)	25 (5.6)	
Gravidity				
1	397 (32.6)	231 (30.1)	166 (37.1)	**0.03**
2	360 (29.6)	230 (29.9)	130 (29.0)	
≥3	459 (37.8)	307 (40.0)	152 (33.9)	
Hb concentration (mg/dL)				
Anemia	97 (8.0)	69 (9.0)	28 (6.3)	0.09
No anemia	1119 (92.0)	699 (91.0)	420 (93.8)	
Random blood glucose (mg/dL)				
≤140	1178 (96.9)	749 (97.5)	429 (95.8)	0.08
>141	38 (3.1)	19 (2.5)	19 (4.2)	
Maternal height (cm)				
<150	304 (25.0)	212 (27.6)	92 (20.5)	**0.006**
≥150	912 (75.0)	556 (72.4)	356 (79.5)	
Number of MMS consumed				
<90	560 (46.1)	374 (48.7)	186 (41.5)	**0.015**
≥90	656 (53.9)	394 (51.3)	262 (58.5)	
Dietary diversity score				
<5	204 (16.8)	135 (17.6)	69 (15.4)	0.32
≥5	1012 (83.2)	633 (82.4)	379 (84.6)	
Smoking exposure				
No	499 (41.0)	296 (38.5)	203 (45.3)	
Passive indoor smoking	717 (59.0)	472 (61.5)	245 (54.7)	**0.02**

*Note*: BMI, calculated as weight in kilograms divided by the square of height in meters. Statistically significant differences indicated in bold.

Abbreviations: BMI, body mass index; GWG, gestational weight gain; MMS, multiple micronutrient supplementation.

^a^ Chi‐square tests.

There were clear associations between participant characteristics and GWG (Table 2). Consumption of ≥90 tablets (OR = 1.33, 95% CI: 1.05–1.69) was more likely to be associated with adequate GWG. Age, pre‐pregnancy BMI, gravidity, height, number of tablets consumed and passive indoor smoking were significantly associated with GWG. For women aged 25 to <30 years, the odds of achieving adequate GWG was 1.33 times the odds for those aged ≥30 years (OR = 1.33, 95% CI: 1.01–1.75). Overweight (OR = 2.31, 95% CI: 1.74–3.07) and obese women (OR = 4.78, 95% CI: 2.98–7.67) were more likely to achieve adequate GWG than those with normal weight. Similarly, women with a height ≥150 cm (OR = 1.47, 95% CI: 1.11–1.94) were more likely to achieve adequate GWG than those who were <150 cm. On the other hand, women with ≥3 gravidities (OR = 0.68, 95% CI: 0.52–0.91) and passive smokers (OR = 0.75, 95% CI: 0.59–0.95) were less likely to have adequate GWG.

**TABLE 2:** Results of univariable logistic regression of characteristics associated with adequate gestational weight gain.

Variables	*P* value	OR (95% CI) adequate GWG
Total MMS consumed	0.028	0.997 (0.994–1.000)
Number of MMS consumed		
<90 tablets		Reference
≥90 tablets	0.01	1.33 (1.05–1.69)
Age (years)		
<20	0.84	1.05 (0.62–1.77)
20 to <25	0.27	1.18 (0.07–1.62)
25 to <30	0.04	1.33 (1.01–1.75)
≥30		Reference
Education		
Junior high school	0.42	0.84 (0.56–1.26)
Senior high school	0.10	0.75 (0.53–1.06)
Diploma		Reference
University	0.12	1.24 (0.94–1.64)
Occupation		
Not working		Reference
Working	0.05	1.33 (1.00–1.78)
Pre‐pregnancy BMI		
Underweight (<18.5)	0.17	0.74 (0.49–1.13)
Normal (18.5 to <25)		Reference
Overweight (25 to <30)	<0.001	2.31 (1.74–3.07)
Obesity (>30)	<0.001	4.78 (2.98–7.67)
Time of MMS introduction		
First trimester		Reference
Second trimester	0.77	0.96 (0.75–2.08)
Third trimester	0.47	1.29 (0.71–2.08)
Gravidity		
1		Reference
2	0.10	0.78 (0.58–1.05)
≥3	0.01	0.68 (0.52–0.91)
Hb concentration (g/dL)		
Anemia	0.09	0.67 (0.42–1.06)
No anemia		Reference
Random blood glucose (mg/dL)		
≤140		Reference
>141	0.09	1.74 (0.91–3.33)
Height (cm)		
<150		Reference
≥150	0.006	1.47 (1.11–1.94)
Dietary diversity score		
<5	0.32	0.85 (0.62–1.17)
≥5		Reference
Smoking exposure		
No		Reference
Passive indoor smoking	0.02	0.75 (0.59–0.95)

*Note*: BMI, calculated as weight in kilograms divided by the square of height in meters. Reference = adequate GWG.

Abbreviations: BMI, body mass index; CI, confidence interval; GWG, gestational weight gain; MMS, multiple micronutrient supplementation; OR, odds ratio.

**FIGURE 2:**
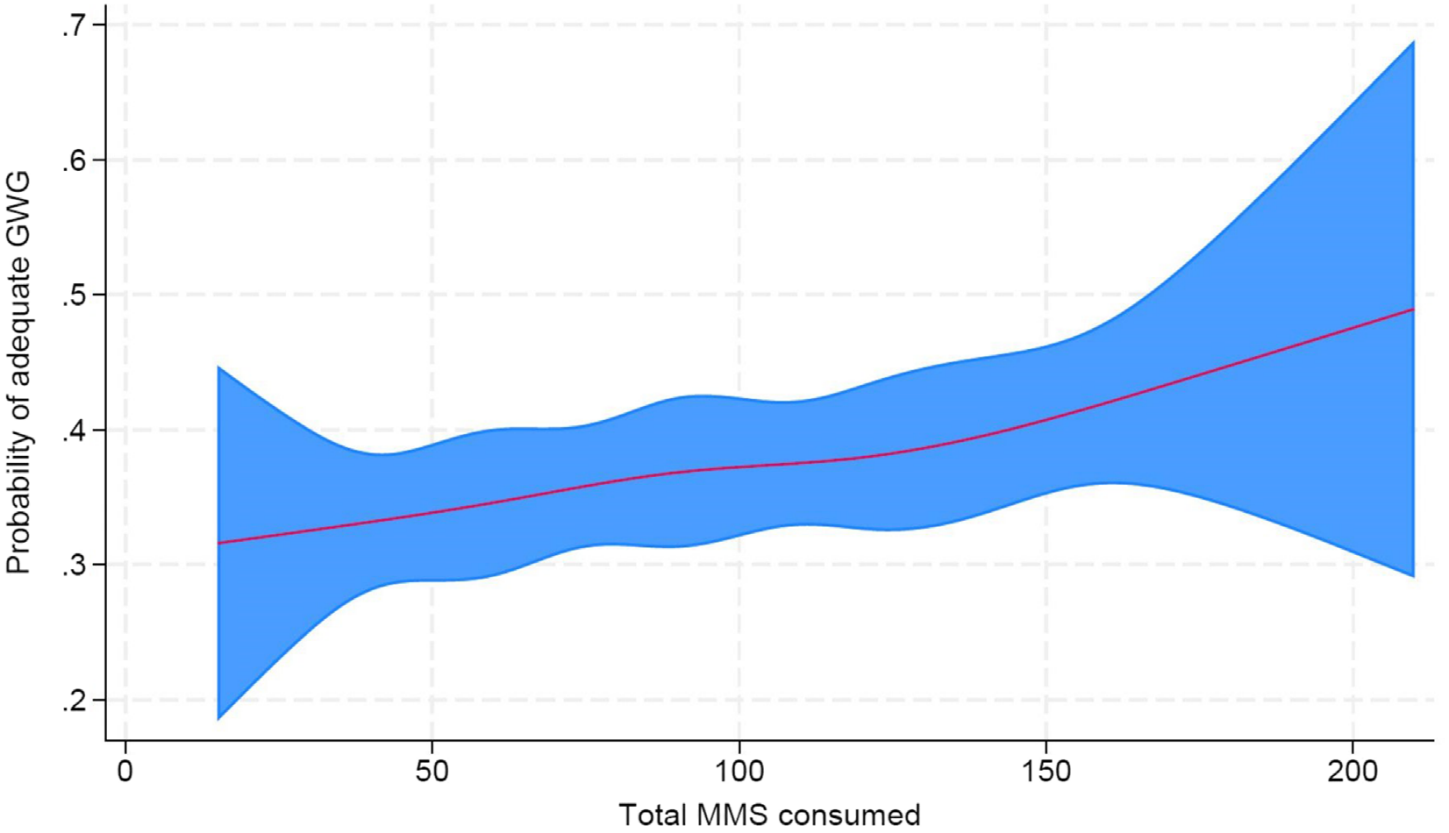
Restricted cubic spline regression of the probability of adequate gestational weight gain (GWG) according to the total number of multiple micronutrient supplementation (MMS) consumed. The probability and its 95% confidence interval (CI) are shown by the red line and shaded area. The curve indicates a higher likelihood of adequate GWG with increased MMS consumption.

We subsequently conducted multivariable logistic regression with covariates having *P* < 0.05 to assess for potential confounding factors of the effect of MMS consumption on GWG adequacy. The effect of MMS consumption was not substantially altered by pre‐pregnancy BMI, height, smoking exposure or any of the other potential confounders (Table S1). Therefore, we presented the unadjusted results as the final result. In addition, we observed a linear relation between the independent variable with GWG (Figure 2).

## 4 DISCUSSION

This study found that prevalence of inadequate GWG based on IOM recommendations in the study population was high. Only one‐third of women achieved adequate GWG. The total number of MMS consumed was significantly associated with an increased likelihood of gaining adequate weight during pregnancy independent of pre‐pregnancy BMI and time of MMS introduction. No other potential confounders materially affected the association between MMS consumption and GWG.

These findings are consistent with an IPD meta‐analysis study that used data from 24 LMIC's that showed that only 22% of pregnant women had adequate GWG.^19^ Similarly, a systematic review and meta‐analysis including studies from several continents found that only 20% of women had adequate GWG,^9^ and also a study in Bangladesh revealed that only 26% of women had adequate GWG.^20^ In the latter study, most women had normal pre‐pregnancy BMI but still did not achieve adequate GWG. Similar data have been reported in a systematic review on data from sub‐Saharan Africa.^21^ found that most pregnant women with normal weight did not meet the minimum recommended GWG based on IOM guidelines. Various factors that could influence GWG may explain these results, including cultural, family, living conditions, genetics, ethnicity, comorbidities and energy balance.^22^ Therefore, counseling on nutritious diets by health care providers, and communication with pregnant women on the first ANC visit about the importance of having adequate GWG is key. Together with regularly monitoring of weight gain over the next visits to address potential challenges can ensure optimal weight gain.^23^

The total number of MMS tablets consumed in our study was significantly associated with an increase in GWG. This finding was in line with an IPD meta‐analysis in LMICs, which showed that MMS resulted in a greater percentage of GWG adequacy and higher GWG at delivery.16 Similarly, two studies conducted in Tanzania on HIV‐negative and HIV positive women found that MMS increased GWG.^24^, ^25^ A study done by Liu et al. in Tanzania found that MMS increased GWG and reduced the risk of severely inadequate and inadequate GWG.^26^ This association was not confirmed in a study by Ramakrishnan et al., who found that MMS did not increase weight gain during pregnancy, but was associated with greater postpartum weight retention among overweight women.^27^ Also, a study done in Ghana found that there was no difference on GWG in pregnant women who consumed MMS and iron or iron‐folic acid.^28^ The possible explanation for these inconsistencies could be the variation in characteristics of the study participants, environmental and social conditions and the dose of MMS given. Particularly in the Tanzanian trials, much higher intakes than the recommended daily allowance were present for vitamin C and several B vitamins, ranging from 6 to 10 times above RDA, and twice the RDA for vitamin E. In contrast, the corresponding vitamin doses in the Ghanaian and Mexican trials were only one to two times the RDA. Our results showed that consuming the provision of the standard UNIMMAP MMS, providing doses of 10 vitamins and five minerals designed to be closed to the RDA, increases the likelihood of gaining sufficient weight during pregnancy. Pregnant women especially in LMIC's therefore should be educated on the benefit of healthy diet and the importance of micronutrients during pregnancy and encouraged to take MMS and nutritious food to meet their nutritional requirements. Having an optimal weight status and ensuring a healthy diet before conception obviously creates the best start, reducing the risk of micronutrient deficiencies during pregnancy.

Even though in many cases supplementation does not start until women are pregnant, there are some explanations why MMS intake started can increase GWG. First, the supplement contains essential micronutrients like vitamin C, B vitamins, and iron, which can directly support fetal growth development and result in higher GWG.^29^, ^30^, ^31^, ^32^ Second, MMS contains antioxidants like vitamins C and E, which may help to mitigate pregnancy‐related oxidative stress^33^ and potentially reduce the risk of low birth weight and preterm birth.^34^ Third, MMS can support immune function of the mother, thereby reducing the risk of infections and any possible negative impacts on maternal weight.^35^, ^36^ Fourth, MMS can increase appetite and satiety, which leads to increased food intake and potentially a more adequate nutrient intake meeting the nutrient needs of pregnancy.^37^, ^38^

Notably, the current WHO BMI cutoff point, mainly based on data from developed countries,^6^ does not cover the full range of pre‐pregnancy BMI^39^, ^40^, ^41^ and may consequently underestimate the effect of MMS on GWG. Clearly, the current WHO BMI cutoff values are higher than Asian BMI standards for normal weight, overweight and obese, making it challenging to universally apply IOM guidelines particularly in LMIC's. Hence, the WHO is advocating for the development of global GWG standards applicable across all pre‐pregnancy BMI levels and geographic locations aiming to define optimal GWG ranges and reduce the risk of adverse maternal and infant health.

The strength of our study was the longitudinal data collection approach in community health care setting in which exposure and outcome measures were prospectively assessed, except for pre‐pregnancy weight. In addition, the study was conducted in the context of the community implementation of MMS the standard health care system allowing for the evaluation of MMS intake in real life setting. The large heterogeneity in the start of MMS as well as the total intakes over the course of pregnancy allowed for detailed assessment of their possible impact on outcomes. Our study also had some limitations: (1) Pre‐pregnancy weight relied on self‐reporting, potentially introducing bias in the classification of the pre‐pregnancy BMI. A published study indicated that self‐reported pre‐pregnancy weight tend to underestimate BMI and thus overestimate GWG.^42^ (2) This data collection was conducted in a small town, potentially limiting generalizability of the findings to other regions, particularly rural areas in Indonesia. (3) The gestational age was determined based on the last day of menstruation instead of an early ultrasound assessment as the gold standard. (4) The total number of MMS consumed was notably influenced by the timing of the first antenatal visit.

## 5 CONCLUSIONS

Our findings indicate that a considerable proportion of pregnant women in Indonesia do not achieve adequate GWG, regardless of pre‐pregnancy BMI. Our results indicate that ensuring intake of the official recommended dose of MMS during pregnancy in Indonesia (>90 tablets) may substantially increase the chance of achieving adequate GWG albeit that optimal supplementation levels need further research investment. Yet, based on these results, advice on the provision of MMS, consumption and appropriate GWG should be incorporated as a part of ANC services for every pregnant woman. Future studies should focus on studying the effect of MMS on GWG when starting already during pre‐conception and continued during pregnancy and also on assessing the number of tablets need to be consumed to achieve best outcomes for mother and child.

## AUTHOR CONTRIBUTIONS

S.M.B, E.v.d.B, and H.G conceptualized and designed the study. S.M.B handled data validation and draft manuscript writing. S.M.B and H.G performed data analysis and visualization. All authors reviewed, edited and approved the final version of the manuscript.

## FUNDING INFORMATION

Indonesia Endowment Funds for Education (LPDP).

## CONFLICT OF INTEREST STATEMENT

The authors have no conflict of interests to declare.

## Supporting information


**Table S1.** Results of multivariable logistic regression of characteristics associated with adequate gestational weight gain.

## Data Availability

Data used are available from the corresponding author upon reasonable request.
