# Supplementary material for: Prenatal multiple micronutrient supplementation is associated with improved maternal gestational weight gain: A prospective longitudinal study in Parepare, Indonesia
Source: Int J Gynaecol Obstet. 2025 Jul 19;172(1):588–95. doi: 10.1002/ijgo.70389 (PMC12724044; doi:10.1002/ijgo.70389)
Supplement: Supplementary file 1 — Table S1. Results of multivariable logistic regression of characteristics associated with adequate gestational weight gain. [file IJGO-172-588-s001.docx]

Supplementary Table S1. Results of multivariable logistic regression of characteristics associated with adequate gestational weight gain

| **Variables** | **P Value** | **OR (95% CI)**  **adequate GWG** | **AOR (95% CI)**  **adequate GWG** |
| --- | --- | --- | --- |
| Total MMS consumed | 0.028 | 0.997 (0.994-1.000) |  |
| Number of MMS consumed |  |  |  |
| <90 tablets |  | Reference |  |
| ≥90 tablets | 0.01 | 1.33 (1.05-1.69) |  |
| Age (y) |  |  |  |
| < 20 | 0.84 | 1.05 (0.62-1.77) | 0.09 (0.64-1.85) |
| 20 to <25 | 0.27 | 1.18 (0.07-1.62) | 1.21 (0.88-1.65) |
| 25 to <30 | 0.04 | **1.33 (1.01-1.75)** | **1.35 (1.02-1.79)** |
| ≥30 |  | Reference |  |
| Prepregnancy BMI |  |  |  |
| Underweight (<18.5) | 0.17 | 0.74 (0.49-1.13) | 0.74 (0.49-1.13) |
| Normal (18.5 to <25) |  | Reference |  |
| Overweight (25 to <30) | <0.001 | **2.31 (1.74-3.07)** | **2.26 (1.70-3.01)** |
| Obesity (>30) | <0.001 | **4.78 (2.98-7.67)** | **4.71 (2.94, 7.56)** |
| Gravidity |  |  |  |
| 1 |  | Reference |  |
| 2 | 0.10 | 0.78 (0.58-1.05) | 0.77 (0.57-1.03) |
| ≥3 | 0.01 | **0.68 (0.52-0.91)** | **0.69 (0.52-0.91)** |
| Height (cm) |  |  |  |
| < 150 |  | Reference |  |
| ≥150 | 0.006 | **1.47 (1.11-1.94)** | **1.48 (1.12-1.95)** |
| Smoking Exposure |  |  |  |
| No |  | Reference |  |
| Passive indoor smoking | 0.02 | **0.75 (0.59-0.95)** | **0.76 (0.60-0.97)** |

Reference = Adequate GWG
